# Gold-Catalyzed Addition of β-Ketoesters to Alkenes: Influence of Electronic and Steric Effects in the Reaction Outcome

**DOI:** 10.3390/molecules23030629

**Published:** 2018-03-10

**Authors:** Agustina La-Venia, Mirta P. Mischne, Ernesto G. Mata

**Affiliations:** Instituto de Química Rosario, Facultad de Ciencias Bioquímicas y Farmacéuticas, Universidad Nacional de Rosario-CONICET, Suipacha 531, Rosario S2002LRK, Argentina; lavenia@iquir-conicet.gov.ar

**Keywords:** gold catalysis, hydroalkylation, β-ketoesters, olefins

## Abstract

The gold-catalyzed intermolecular hydroalkylation of olefins with β-ketoesters represents a conceptually attractive and useful synthetic tool; however, it has been scarcely applied, remaining a challenge for chemists. The aim of the current study was to investigate the addition of these 1,3-diketo-compounds to alkenes under gold catalysis conditions, in order to establish the electronic and steric effects of the alkenyl substrates in the reaction outcome. The screening of different catalyst systems and diverse olefins enabled defining the alkenyl requirements and the best reaction conditions to efficiently achieve the coupled products.

## 1. Introduction

In recent years, the application of homogeneous gold catalysis has become a very active area of research [1,2,3,4,5,6,7,8,9,10,11,12,13]. Gold is considered a “soft” metal, thus it is an excellent carbophilic activator avoiding high oxophilicity, which is exhibited by most of the Lewis acids. Therefore, gold selectively activates unsaturated carbon–carbon bonds (alkynes, alkenes, and allenes) towards nucleophilic attack, catalyzing a large number of organic transformations with high efficiency under a broad scope of reaction conditions (oxygen, water, and alcohols are usually tolerated) [14,15,16,17,18,19]. The gold-promoted nucleophilic additions onto alkynes and allenes have been widely explored for the generation of both new C-heteroatom (N, S, O) bonds and new carbon–carbon bonds [20,21,22,23,24,25,26]. In the last few years, gold-mediated activation of alkenes has been developed as well, but in less extension as expected, due to their lower reactivity comparing with the alkynyl and allenyl counterparts [27,28,29,30,31]. In particular, gold catalysis applied to unactivated olefins presents a limited scope of conditions and reagents, and is mainly referred to heteroatom nucleophilic addition, which, in most of the cases, leads to the formation of heterocyclic structures [32,33,34,35,36,37,38]. Indeed, the formation of new carbon–carbon bonds achieved by gold catalysis from alkenyl systems remains scarcely investigated [39,40,41,42]. Specifically, the gold-mediated hydroalkylation of olefins using 1,3-dicarbonyl systems, which represents an atom-economic alternative to the classic nucleophilic alkylation, has been barely explored [43,44,45,46]. This conceptually attractive synthetic approach presents only a few examples of intermolecular addition of 1,3-diketo-compounds to alkenes, mainly limited to the use of electron-rich alkenes and Au(III) catalysts [43,45,47].

In this context, we decided to investigate the scope and limitations of gold catalysis for the intermolecular addition of simple β-ketoesters onto diverse substituted alkenes, attempting to establish the influence of steric and electronic factors on the course of this process.

## 2. Results and Discussion

**Scheme 1:**
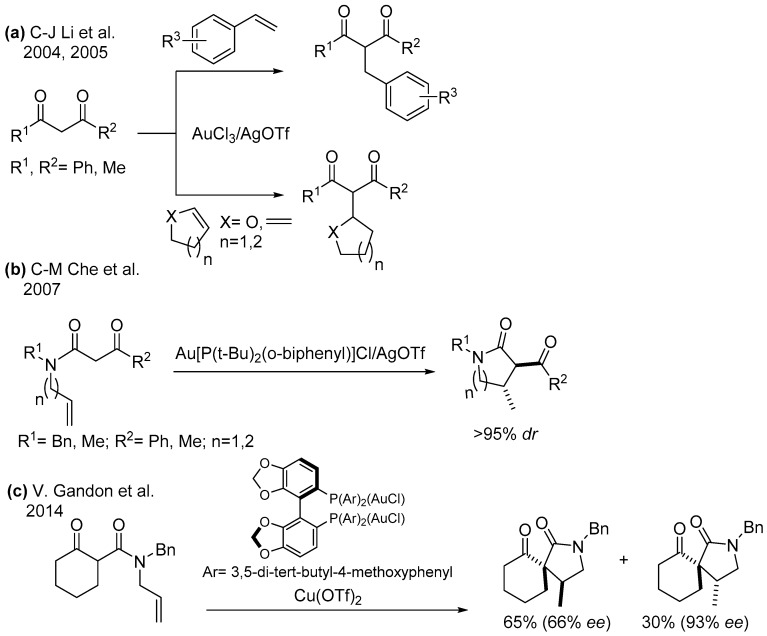
Gold-catalyzed hydroalkylation of alkenes with 1,3-dicarbonyl compounds. (**a**) Intermolecular version with 1,3-diketones. (**b**) and (**c**) Intramolecular version with β-ketoamides.

As previously exposed, the reported examples of gold catalysis applied to the chemical transformation under study refer to the coupling of 1,3-diketones either with aryl-conjugated alkenes [45] or with electron-rich cyclic alkenes [43,47] in the presence of cationic Au(III) (Scheme 1a). Particularly interesting are the intramolecular versions of this process that enable the use of alternative dicarbonyl systems, for instance β-ene-1,3-diketoamides, which afford cyclic lactams (Scheme 1b) [46]. Moreover, in the presence of (*R*)-DTBM-SEGPHOS(AuCl)_2_ and Cu(OTf)_2_, the asymmetric version of this ene-β-ketoamide cyclization was achieved (Scheme 1c) [40]. The key modification in this enantioselective hydroalkylation was the use of copper chloride scavengers [48,49].

It is apparent from these reports that the use of β-ketoesters as the dicarbonyl starting material has been underexplored. In fact, both Li et al. [45] and Che et al. [46] specifically highlighted that 1,3-dicarbonyl substrates incorporating ester functionalities failed to proceed to the corresponding C–C bond formation under gold catalysis, probably due to decomposition of these ester-functionalized starting materials in the presence of high Lewis acidic reagents [45]. In addition, the intramolecular alternative using these kinds of diketo moieties has not been efficiently achieved either. Recently, Gandon and coworkers reported a gold(I)-mediated cyclization via an intramolecular hydroalkylation of an internal diene by a β-ketoester [44]. However, optimized reaction conditions were accomplished using Bi(OTf)_3_/TfOH as an alternative catalytic system due to the fact that extensive decomposition of starting material was observed using gold catalysts. Some other studies were carried out trying to expand the hydroalkylation of olefins with β-ketoesters mediated by auric cations; however, they were clearly unsuccessful [50]; furthermore, in some conditions, the gold catalysts exhibited an unusual oxophilic behavior [51].

Taking into account this literature review, to achieve our objective, we decided firstly to test the reaction between β-ketoesters (**1a** and **1b**) and *p*-methylstyrene (**2a**) to obtain the corresponding coupling products **3a** and **3b** (Table 1). Different reaction conditions, mainly involving changes in the catalyst mixture and temperature, were evaluated. For ethyl acetoacetate (**1a**), the use of a AuCl_3_/AgSbF_6_ mixture (5 mol %/15 mol %) was the most efficient catalyst system, leading to **3a** in 75% yield (Entry 1). The product was obtained as an inseparable mixture of two diastereoisomers in a 1:1 ratio, identified and quantified by ^1^H-NMR spectra and by HPLC. The use of AgOTf as co-catalyst (Entry 2) provided similar results, whereas the use of Au(I) catalyst (Entry 3) caused a significant decrease in yield. The addition of CuCl_2_ to avoid gold reduction resulted in a slight decrease in the reaction yield (Entry 4) [52]. To evaluate the protic triflic acid (TfOH) effect, this acid was added as the single promoter (Entry 5) [53]. The expected product **3a** was obtained but with a notable decreased yield (20%). Similarly, the use of AgOTf as the only catalyst was also tested, but the reaction did not proceed at all, recovering the starting materials (Entry 6). These latter results proved that auric triflate is the predominant catalytic specie involved in the hydroalkylation under study. On the other hand, changing the dicarbonyl substrate by *tert*-butyl acetoacetate (**1b**) gave a complex mixture of unidentified compounds, probably derived from self-condensation of the alkene and β-ketoester hydrolysis in the acidic media (Entry 7).

With these optimized conditions in hand, we proceeded to investigate the scope and limitations of the process testing different olefin partners. ^1^H-NMR spectroscopic analysis of the crude reaction mixtures showed that, along with the presence of alkylated products **3** and starting materials **1a** and **2**, signals corresponding to self-condensation olefin polymeric by-products [54,55] could be identified (Table 2).

A detailed examination of the results indicates that the reaction under study is very sensitive to structural and electronic features of the alkene. In the case of *para*-substituted styrene derivatives (**2a**–**i**), the best results have been achieved for those substrates having both weak electron-donating such as alkyl groups (**2a**–**c**, Entries 1–3) and weak electro-withdrawing substituents (**2d**, Entry 4), leading to the adducts **3a**–**d** up to 75% yield. In the case of other aromatic substrates with stronger electron-donating groups (**2e**–**g**), the corresponding products were detected but with considerable decrease in the yields and along with large amounts of polymeric material (Entries 5 and 6), except for **2g** (Entry 7). These results revealed an efficient gold-promoted alkene-activation, except for the most bulky substituted styrene (*R*^2^ = O*t*Bu); however, unfortunately, the polymerization process was predominant. Interestingly, those substrates with strong electron-withdrawing substituents (**2h**, *R*^2^ = CO_2_Me and **2i**, *R*^2^ = Br, Entries 8 and 9) did not provide the desired products and neither the possible products of polymerization, recovering both starting materials, which could be justified by an ineffective olefin coordination with the metal. A similar tendency was observed for *ortho*-substituted styrene derivatives **2j** and **2k**, which did not generate the desired addition products, regardless of their electronic properties, recovering in both cases the unreacted starting materials (Entries 10 and 11). Though, in the case of alkene **2k** (Entry 11), a small amount of polymerization product was observed, demonstrating a poor activation of the olefin by the Au(III) [56,57], probably due to steric hindrance of the substituent in *ortho* position in comparison to the *para* analogue **2a**. As expected, the non-aromatic alkenes or alkenes structurally different to styrene, remained unchanged under the optimized conditions (Entries 12–14), which clearly indicated that the cationic gold failed to promote their activation.

Taking into account these results, we can remark about two major difficulties for the broad application of this synthetic tool. According to the most commonly proposed mechanism based on the cationic gold activation of alkene species followed by methylene nucleophilic attack, the first difficulty is related to the activation of alkenes by coordination with the active gold species. In order to achieve such gold coordination, olefins must present sufficient electron density, restricting the type of alkene able to participate in the hydroalkylation. This limitation becomes evident in those alkenes with low electron density, which remained unreacted in the presence of the auric cation (**2h**–**j** and **2l**–**n**).

The second drawback of the reaction arises from competition with the self-addition of the olefins. Electron rich olefin can coordinate with the cationic gold, but the presence of free unactivated olefin in the reaction medium can also efficiently act as a nucleophile. Thus, self-addition occurs between the metal-coordinated olefin (electrophilic) and the free olefin (nucleophilic), generating the polymeric by-product. There is a competition between both potential nucleophiles, high-electron density olefins and the corresponding enol of 1,3-dicarbonyl compounds. The nucleophilic enolate can be generated by the presence of metal co-catalyst, such as Cu or can be present directly in the dicarbonyl equilibrium. In the case of β-ketoesters, only a small proportion is present as the enol form (8% in DCM) [58]; therefore, high electron density olefins are better nucleophiles, generating mainly polymerization products during catalysis. In contrast, reactions involving diketones instead of β-ketoesters are more efficient for the hydroalkylation of alkenes since these diketo compounds are mostly in their enol form (81% in DCM).

Under these premises, we carried out further screening conditions to some promising substrates (**2e** and **2f**). It can be assumed that reduction of olefin self-addition would increase the efficiency of the hydroalkylation reaction. To achieve the limitation of this undesired reaction, various strategies were tested. The first attempts that involved the incorporation of base to the reaction mixture in order to shift the keto/enol equilibrium did not proceed as expected. For instance, the addition of *t*BuOK led to a slight increase of coupling while amine bases poisoned the catalyst. Fortunately, slow alkene addition at low temperature showed promising effects. Moreover, when the decrease in temperature was combined with a simple exchange of co-catalyst (AgOTf instead of AgSbF_6_), **3f** was obtained with a considerably higher yield (25%) (Entry 6, Table 2). Similar results were observed for substrate **2d** (*R*^2^ = CH_2_Cl), affording **3d** with a 35% yield (Entry 4, Table 2).

## 3. Materials and Methods

### 3.1. General Information

Chemical reagents were purchased from commercial suppliers and used without further purification, unless otherwise noted. Solvents were analytical grade or were purified by standard procedures prior to use. Reactions requiring inert atmosphere were carried out under a high-purity dry nitrogen atmosphere. Solvents from these reactions were transferred with syringe under high-purity dry nitrogen pressure. Yields were calculated for material judged homogeneous by thin layer chromatography (TLC) and nuclear magnetic resonance (^1^H-NMR). All reactions were monitored by thin layer chromatography performed on silica gel 60 F254 pre-coated aluminum sheets, visualized by a 254 nm UV lamp, and stained with an ethanolic solution of 4-anisaldehyde. Column flash chromatography was performed using silica gel 60 (230–400 mesh).

### 3.2. Instrumental and Physical Data

1H-NMR spectra were recorded in a Bruker Avance spectrometer (Bruker Analytik GmbH, Karlsruhe, Germany) at 300 MHz, in CDCl_3_ with tetramethylsilane (TMS) as internal standard (0 ppm). ^13^C-NMR spectra were recorded on the same apparatus at 75 MHz with CDCl_3_ as solvent and reference (76.9 ppm). Chemical shifts (δ) are reported in ppm upfield from TMS and coupling constants (*J*) are expressed in Hertz. The following abbreviations are used to indicate the multiplicities: s = singlet, d = doublet, t = triplet, q = quartet, m = multiplet, bs = broad singlet.

IR spectra were obtained using a Shimadzu, Prestige–21 FT-IR spectrometer (Shimadzu, Kioto, Japan), wavelengths are informed in cm^−1^, and only partial spectral data are listed.

High resolution mass spectra (HRMS) were recorded on a Bruker micrOTOF-Q II spectrometer obtained on a Q-TOF mass spectrometer and detection of the ions was performed in electrospray ionization, positive ion mode.

### 3.3. Synthetic Procedures

Gold-catalyzed hydroalkylation: A mixture of AuCl_3_ (15.2 mg, 0.05 mmol, 5 mol %) and AgSbF_6_ (51.5 mg, 0.15 mmol, 15 mol %) in anhydrous DCM (2 mL) under N_2_ atmosphere was stirred at room temperature for 2 h. The β-ketoester, ethyl acetoacetate **1a**, (0.13 mL, 1 mmol) was then added to the catalysts solution previously formed, followed by the addition of the solution of the corresponding alkene **2** (1.5 mmol, 1.5 eq.) in anhydrous DCM (3 mL) with a syringe-pump during 5 h at room temperature or at 0 °C. The reaction mixture was further stirred at room temperature overnight under N_2_ atmosphere. After that time, the solvent was evaporated and the reaction crude was purified by column chromatography on silica gel (eluent: Hexane-AcOEt with increasing polarity) to afford addition products **3** (1:1 diastereoisomeric mixture). Note: the AuCl_3_ catalyst must be weighed under a nitrogen cone because of its high hygroscopicity.

### 3.4. Analytical Data of Individual Compounds

*Ethyl 2-acetyl-3-(p-tolyl)butanoate* **3a**. Yield: 75% of colorless oil (inseparable mixture with diastereoisomeric ratio 1:1). IR (Film) (cm^−1^): 2964, 2932, 1743 (νCO), 1717 (νCO), 1513, 1177. ^1^H-NMR (CDCl_3_, 300 MHz): δ 7.09 (8H, bs, ArH), 4.22 (2H, q, *J* = 7.2 Hz, -OC*H*_2_CH_3_), 3.90 (2H, q, *J* = 7.1 Hz, -OC*H*_2_CH_3_), 3.76 (1H, d, *J* = 11.0 Hz, H-2), 3.71 (1H, d, *J* = 10.9 Hz, H-2), 3.56–3.45 (2H, m, H-1′), 2.30 (6H, s, ArCH_3_)*, 2.29 (3H, s, H-4)*, 1.94 (3H, s, H-4)*, 1.31–1.27 (6H, m, H-2′ and -OCH_2_C*H*_3_), 1.21 (3H, d, *J* = 6.9 Hz, H-2′), 0.97 (3H, t, *J*= 7.2 Hz, -OCH_2_C*H*_3_). ^13^C NMR (CDCl_3_, 75 MHz): δ 202.5 (C, C-3), 202.4 (C, C-3), 168.6 (C, C-1), 168.1 (C, C-1), 140.1 (C, Ar), 139.9 (C, Ar), 136.4 (C, Ar), 136.2 (C, Ar), 129.3 (2 CH, Ar), 129.0 (2 CH, Ar), 127.2 (2 CH, Ar), 127.1 (2 CH, Ar), 67.6 (CH, C-2), 67.1 (CH, C-2), 61.3 (CH_2_, -O*C*H_2_CH_3_), 61.0 (CH_2_, -O*C*H_2_CH_3_), 39.6 (CH, C-1′), 39.3 (CH, C-1′), 29.7 (CH_3_, C-4), 29.4 (CH_3_, C-4), 20.9 (2 CH_3_, Ar-CH_3_), 20.6 (CH_3_, C-2′), 20.3 (CH_3_, C-2), 14.0 (CH_3_, -OCH_2_*C*H_3_), 13.6 (CH_3_, -OCH_2_*C*H_3_). HRMS (ESI) *m*/*z* calcd. for C_15_H_20_NaO_3_ [M + Na]^+^ 271.1305, found 271.1302.


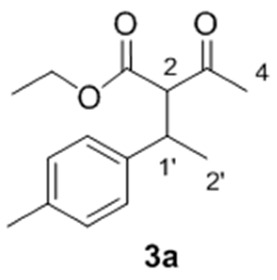


*Ethyl 2-acetyl-3-(4-ethylphenyl)butanoate* **3b**. Yield: 50% of colorless oil (inseparable mixture with diastereoisomeric ratio 1:1). IR (Film) (cm^−1^): 2965, 2932, 2873, 1744 (νCO), 1716 (νCO), 1513, 1176. ^1^H-NMR (CDCl_3_, 300 MHz): δ 7.11 (8H, bs, ArH), 4.22 (2H, q, *J* = 7.2 Hz, -OC*H*_2_CH_3_), 3.89 (2H, q, *J* = 7.1 Hz, -OC*H*_2_CH_3_), 3.77 (1H, d, *J* = 11.0 Hz, H-2), 3.71 (1H, d, *J* = 11.0 Hz, H-2), 3.59–3.46 (2H, m, H-1′), 2.60 (4H, q, *J* = 7.5 Hz, H-1″), 2.29 (3H, s, H-4), 1.93 (3H, s, H-4), 1.31-1.17 (15H, m, H-2′, H-2″ and -OCH_2_C*H*_3_)*, 0.94 (3H, t, *J* = 7.1 Hz, H-2″)*. ^13^C NMR (CDCl_3_, 75 MHz): δ 202.5 (2 C, C-3), 168.5 (C, C-1), 168.1 (C, C-1), 142.6 (2 C, Ar), 140.2 (C, Ar), 140.1 (C, Ar), 128.0 (2 CH, Ar), 127.7 (2 CH, Ar), 127.2 (2 CH, Ar), 127.1 (2 CH, Ar), 67.6 (CH, C-2), 67.0 (CH, C-2), 61.3 (CH_2_, -O*C*H_2_CH_3_), 60.9 (CH_2_, -O*C*H_2_CH_3_), 39.6 (CH, C-1′), 39.3 (CH, C-1′), 29.7 (CH_3_, C-4), 29.3 (CH_3_, C-4), 28.3 (CH_2_, C-1″), 28.2 (CH_2_, C-1″), 20.5 (CH_3_, C-2′)*, 20.2 (CH_3_, C-2′)*, 15.4 (CH_3_, C-2″)*, 15.3 (CH_3_, C-2″)*, 14.0 (CH_3_, -OCH_2_*C*H_3_), 13.6 (CH_3_, -OCH_2_*C*H_3_). HRMS (ESI) *m*/*z* calcd. for C_16_H_23_NaO_3_ [M + Na]^+^ 285.1461, found 285.1461.


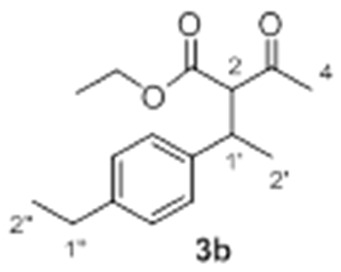


*Ethyl 2-acetyl-3-(4-(tert-butyl)phenyl)butanoate* **3c** Yield: 20% of colorless oil (inseparable mixture with diastereoisomeric ratio 1:1). ^1^H-NMR (CDCl_3_, 300 MHz): δ 7.29 (4H, d, *J* = 8.5 Hz, ArH), 7.15–7.10 (4H, m, ArH), 4.22 (2H, q, *J* = 7.1 Hz, -OC*H*_2_CH_3_), 3.93–3.83 (2H, m, -OC*H*_2_CH_3_), 3.77 (1H, d, *J* = 10.9 Hz, H-2), 3.70 (1H, d, *J* = 11.0 Hz, H-2), 3.57–3.48 (2H, m, H-1′), 2.30 (3H, s, H-4), 1.93 (3H, s, H-4), 1.34–1.22 (27H, m, H-2′, -C(CH_3_)_3_ and -OCH_2_C*H*_3_)*, 0.88 (3H, t, *J* = 7,1 Hz, -OCH_2_C*H*_3_)*. ^13^C NMR (CDCl_3_, 75 MHz): δ 202.5 (2 C, C-3), 168.6 (C, C-1), 168.5 (C, C-1); 149.6 (C, Ar); 149.5 (C, Ar); 139.9 (C, Ar); 139.8 (C, Ar); 127.0 (2 CH, Ar); 126.8 (2 CH, Ar); 125.4 (2 CH, Ar); 125.1 (2 CH, Ar); 67.7 (CH, C-2); 67.0 (CH, C-2); 61.3 (CH_2_, -O*C*H_2_CH_3_); 60.9 (CH_2_, -O*C*H_2_CH_3_); 39.5 (CH, C-1′); 39.3 (CH, C-1′); 34.3 (2 C, -*C*(CH_3_)_3_); 31.2 (6 CH_3_, -C(*C*H_3_)_3_); 29.7 (CH_3_, C-4); 29.3 (CH_3_, C-4); 20.5 (CH_3_, C-2′); 20.1 (CH_3_, C-2’); 14.0 (CH_3_, -OCH_2_*C*H_3_); 13.5 (CH_3_, -OCH_2_*C*H_3_). HRMS (ESI) *m*/*z* calcd. for C_18_H_30_NO_3_ [M + NH_4_]^+^ 308.2226, found 308.2218.


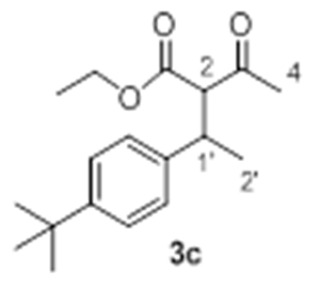


*Ethyl 2-acetyl-3-(4-(chloromethyl)phenyl)butanoate* **3d** Yield: 35% of colorless oil (inseparable mixture with diastereoisomeric ratio 1:1). ^1^H-NMR (CDCl_3_, 300 MHz): δ 7.31 (4H, d, *J* = 7.5 Hz, ArH), 7.23–7.18 (4H, m, ArH), 4.55 (4H, bs, -CH_2_Cl), 4.23 (2H, q, *J* = 7.1 Hz, -OC*H*_2_CH_3_), 3.89 (2H, q, *J* = 7.1 Hz, -OC*H*_2_CH_3_), 3.78 (1H, d, *J* = 10.8 Hz, H-2), 3.73 (1H, d, *J* = 10.9 Hz, H-2), 3.61–3.50 (2H, m, H-1′), 2.30 (3H, s, H-4), 1.96 (3H, s, H-4), 1.31–1.27 (6H, m, H-2′ and -OCH_2_C*H*_3_), 1.23 (3H, d, *J* = 6.9 Hz, H-2′), 0.95 (3H, t, *J* = 7,1 Hz, -OCH_2_C*H*_3_). ^13^C NMR (CDCl_3_, 75 MHz): δ 202.0 (C, C-3), 201.9 (C, C-3), 168.3 (C, C-1), 167.9 (C, C-1), 143.5 (C, Ar), 143.4 (C, Ar), 135.9 (2 C, Ar), 128.8 (2 CH, Ar), 128.6 (2 CH, Ar), 127.7 (2 CH, Ar), 127.6 (2 CH, Ar), 67.2 (CH, C-2), 66.8 (CH, C-2), 61.4 (CH_2_, -O*C*H_2_CH_3_), 61.1 (CH_2_, -O*C*H_2_CH_3_), 45.8 (CH_2_, -*C*H_2_Cl), 45.7 (CH_2_, -*C*H_2_Cl), 39.4 (CH, C-1′), 39.3 (CH, C-1′), 29.7 (CH_3_, C-4), 29.4 (CH_3_, C-4), 20.3 (CH_3_, C-2′), 20.2 (CH_3_, C-2′), 14.0 (CH_3_, -OCH_2_*C*H_3_), 13.6 (CH_3_, -OCH_2_*C*H_3_). HRMS (ESI) *m*/*z* calcd. for C_15_H_23_ClNO_3_ [M + NH_4_]^+^ 300.1366, found 300.1358.


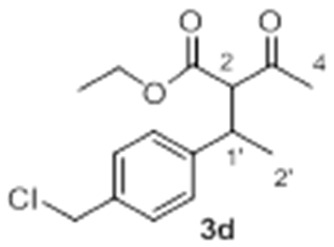


*Ethyl 2-acetyl-3-(4-methoxyphenyl)butanoate* **3f** Yield: 25% of colorless oil (inseparable mixture with diastereoisomeric ratio 1:1). ^1^H-NMR (CDCl_3_, 300 MHz): δ 7.14 (2H, d, *J* = 6.0 Hz, ArH), 7.11 (2H, d, *J* = 6.0 Hz, ArH), 6.82 (4H, dd, *J* = 8.7, 0.9 Hz, ArH), 4.21 (2H, q, *J* = 7.2 Hz, -OC*H*_2_CH_3_), 3.90 (2H, q, *J* = 7.2 Hz, -OC*H*_2_CH_3_), 3.77 (6H, s, -OCH_3_), 3.74 (1H, d, *J* = 11.1 Hz, H-2), 3.68 (1H, d, *J* = 11.1 Hz, H-2), 3.55-3.44 (2H, m, H-1′), 2.29 (3H, s, H-4), 1.93 (3H, s, H-4), 1.31–1.26 (6H, m, H-2′ and -OCH_2_C*H*_3_), 1.21 (3H, d, *J* = 6.9 Hz, H-2′), 0.98 (3H, t, *J* = 7.2 Hz, -OCH_2_C*H*_3_). ^13^C NMR (CDCl_3_, 75 MHz): δ 202.5 (C, C-3), 202.4 (C, C-3), 168.6 (C, C-1), 168.1 (C, C-1), 158.2 (2 C, Ar), 135.2 (C, Ar), 134.9 (C, Ar), 128.2 (4 CH, Ar), 113.9 (2 CH, Ar), 113.6 (2 CH, Ar), 67.7 (CH, C-2), 67.1 (CH, C-2), 61.3 (CH_2_, -O*C*H_2_CH_3_), 61.0 (CH_2_, -O*C*H_2_CH_3_), 55.1 (CH_3_, -OCH_3_), 55.0 (CH_3_, -OCH_3_), 39.2 (CH, C-1′), 38.9 (CH, C-1′), 29.7 (CH_3_, C-4), 29.3 (CH_3_, C-4), 20.6 (CH_3_, C-2′), 20.3 (CH_3_, C-2′), 14.0 (CH_3_, -OCH_2_*C*H_3_), 13.6 (CH_3_, -OCH_2_*C*H_3_). HRMS (ESI) *m*/*z* calcd. for C_15_H_24_NO_4_ [M + NH_4_]^+^ 282.1705, found 282.1694.


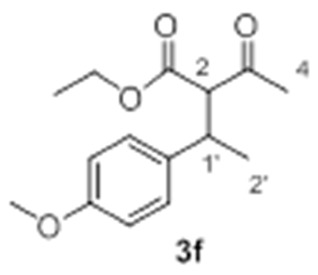


## 4. Conclusions

In conclusion, this study demonstrates that the intermolecular hydroalkylation of alkenes with β-ketoesters catalyzed by cationic gold species can be achieved efficiently by selecting the substrate patterns. It is worthwhile to highlight that, in most of the cases, the β-ketoester substrate (ethylacetoacetate **1a**) exhibited a significant stability to gold catalysis conditions, and only under certain conditions (high temperature, addition of extra acid to the reaction mixture) did its hydrolysis took place. Regarding the alkene requirements, it is concluded that the coupling reaction proceeds efficiently only with those olefinic structures whose electron density is enough to coordinate with the cationic gold species but not too elevated to exhibit predominant nucleophilic character that would lead to the self-addition as the main reaction and, therefore, prevent the reaction with the nucleophilic 1,3-dicarbonyl moiety of the β-ketoester. These requirements limit the scope of suitable alkenes for this synthetic tool. In order to improve yields, low temperature during a very slow addition of the olefin was successfully carried out.

## Figures and Tables

**Table 1:**
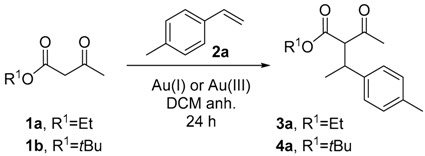
Gold-catalyzed hydroalkylation of *p*-methylstyrene (**2a**) with β-ketoesters **1a** and **1b**.

	1	R^1^	Catalyst System	T	Yield ^1^
1	**1a**	Et	AuCl_3_ (5 mol %) AgSbF_6_ (15 mol %)	rt	75%
2	**1a**	Et	AuCl_3_ (5 mol %) AgOTf (15 mol %)	rt	70%
3	**1a**	Et	AuCl (5 mol %) AgSbF_6_ (5 mol %)	rt	40%
4	**1a**	Et	AuCl_3_ (5 mol %) AgSbF_6_ (15 mol %) CuCl_2_ (10 mol %)	0 °C	60%
5	**1a**	Et	TfOH (2 mol %)	0 °C	20%
6	**1a**	Et	AgOTf (15 mol %)	rt	NR ^2^
7	**1b**	*t*Bu	AuCl_3_ (5 mol %) AgSbF_6_ (15 mol %)	rt	NR ^2^

The reaction was carried out by adding **2a** (1.5 eq.) for 5 h with a syringe pump to the solution of the β-ketoesters **1a** or **1b** (1.0 eq.) and catalyst in dichloromethane, then the reaction mixture was stirred overnight. The addition products **3** were eventually isolated and purified by column chromatography. ^1^ Yield after column chromatography. ^2^ No reaction.

**Table 2:**
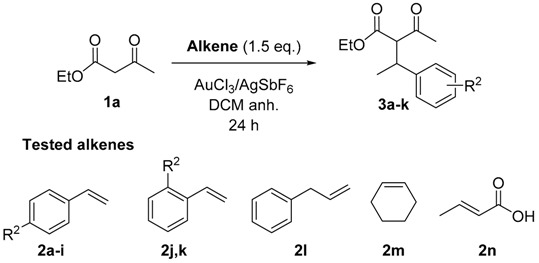
Evaluation of olefin influence in the AuCl_3_/AgSbF_6_–promoted hydroalkylation.

	Alkene	Product ^1^	Recovered Starting Material ^2^	Polymeric by-Products ^3^
1	**2a**, *R*^2^ = *p*-Me	**3a**, 75%	**1a**	Yes
2	**2b**, *R*^2^ = *p*-Et	**3b**, 50%	**1a**	Yes
3	**2c**, *R*^2^ = *p*-*t*Bu	**3c**, 20%	**1a**	Yes
4	**2d**, *R*^2^ = *p*-CH_2_Cl	**3d**, 10% (35%) ^4^	**1a**	Yes
5	**2e**, *R*^2^ = *p*-OAc	**3e**, traces	**1a**	Yes
6	**2f**, *R*^2^ = *p*-OMe	**3f**, 7% (25%) ^4^	**1a**	Yes
7	**2g**, *R*^2^ = *p*-O*t*Bu	**3g**, traces	**1a**	No
8	**2h**, *R*^2^ = *p*-CO_2_Me	NP	**1a** + **2h**	No
9	**2i**, *R*^2^ = *p*-Br	NP	**1a** + **2i**	No
10	**2j**, *R*^2^ = *o*-Br	NP	**1a** + **2j**	No
11	**2k**, *R*^2^ = *o*-Me	NP	**1a** + **2k**	Yes
12	**2l**	NP	**1a** + **2l**	No
13	**2m**	NP	**1a** ^5^	No
14	**2n**	NP	**1a** + **2n**	No

^1^ Yields correspond to final products purified by flash column chromatography. The addition products **3** were generated as inseparable mixtures of diastereoisomers in 1:1 ratio (see Supplementary Materials). NP: No addition product **3** was observed. ^2^ Starting material remained in the reaction mixtures. ^3^ Signals attributed to polymeric by-products were observed by ^1^H-NMR spectra of the reaction mixtures. ^4^ Optimized conditions: AuCl_3_/AgOTf (5/15 mol %), 0 °C. ^5^ **1a** was partially hydrolyzed, **2m** was evaporated during the work up of the reaction.

## Supplementary Materials

The following are available online, Figures S1–4: ^1^H, ^13^C, HSQC, and HMBC NMR spectra of compound **3a** in CDCl_3_, Figures S5–8: ^1^H, ^13^C, HSQC, and HMBC NMR spectra of compound **3b** in CDCl_3_, Figures S9–12: ^1^H, ^13^C, HSQC, and HMBC NMR spectra of compound **3c** in CDCl_3_, Figures S13–16: ^1^H, ^13^C, HSQC, and HMBC NMR spectra of compound **3d** in CDCl_3_, Figures S17–20: ^1^H, ^13^C, HSQC, and HMBC NMR spectra of compound **3f** in CDCl_3_.
