# Supplementary material for: Gold-Catalyzed Addition of β-Ketoesters to Alkenes: Influence of Electronic and Steric Effects in the Reaction Outcome
Source: Molecules. 2018 Mar 10;23(3):629. doi: 10.3390/molecules23030629 (PMC6017100; doi:10.3390/molecules23030629)
Supplement: Supplementary file 1 [file molecules-23-00629-s001.pdf]

Supplementary Materials: Gold-catalyzed addition of β-ketoesters to alkenes. Influence of electronic and steric effects in the reaction outcome.

Agustina La-Venia, Mirta P. Mischne, and Ernesto G. Mata

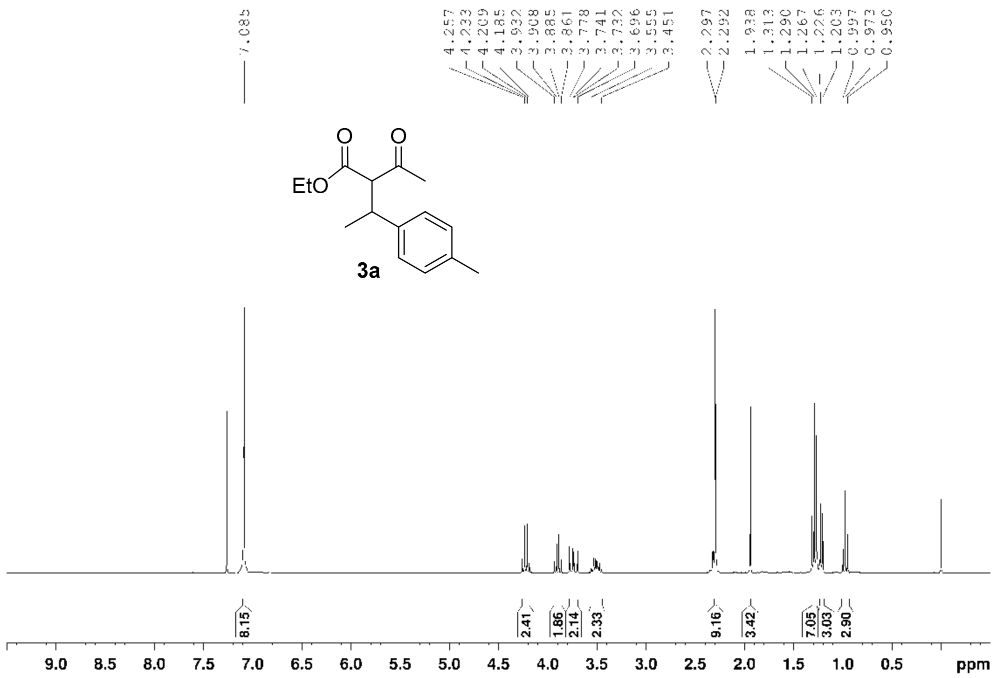

Figure S1. ^{1}H NMR of compound **3a** in $CDCl_3$.

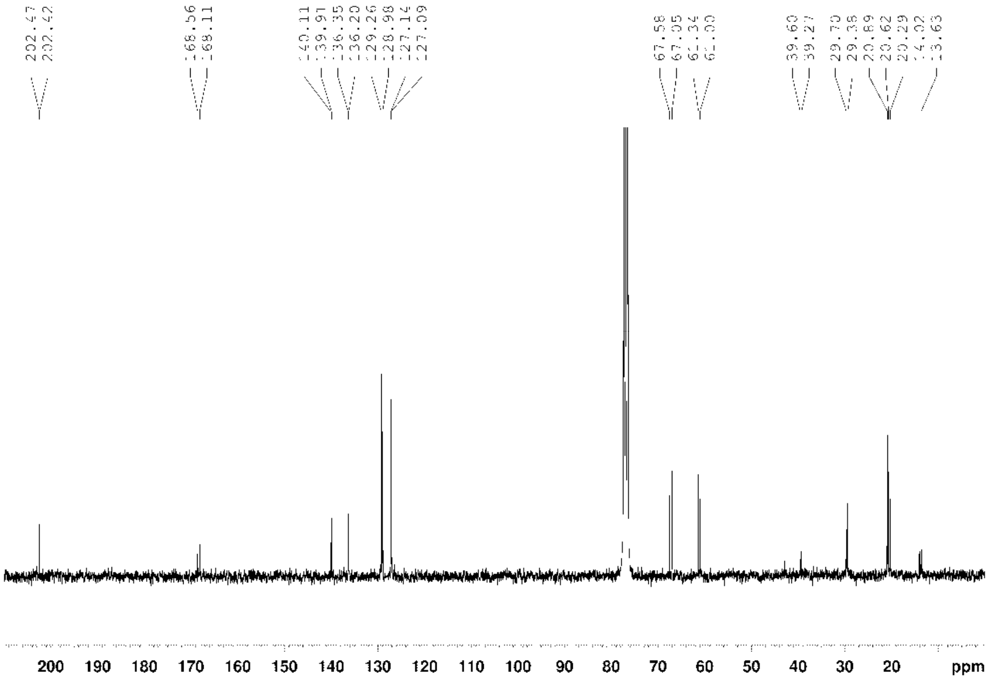

Figure S2. ^{13}C NMR of compound **3a** in $CDCl_3$.

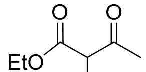

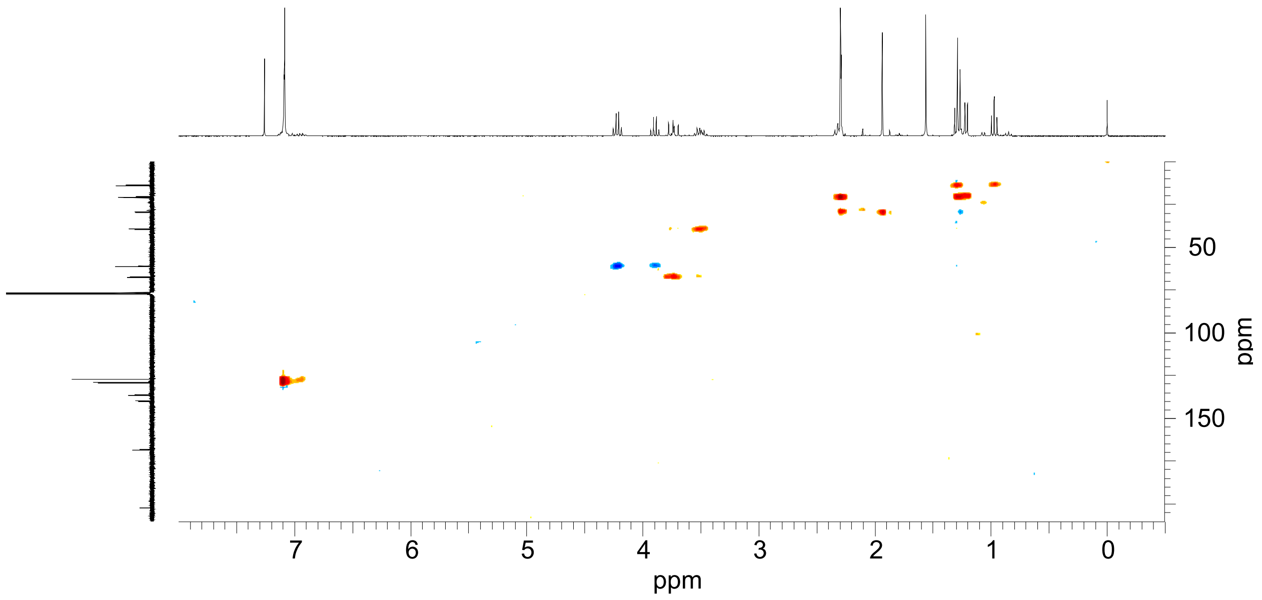

Figure S3. HSQC NMR of compound **3a** in $CDCl_3$.

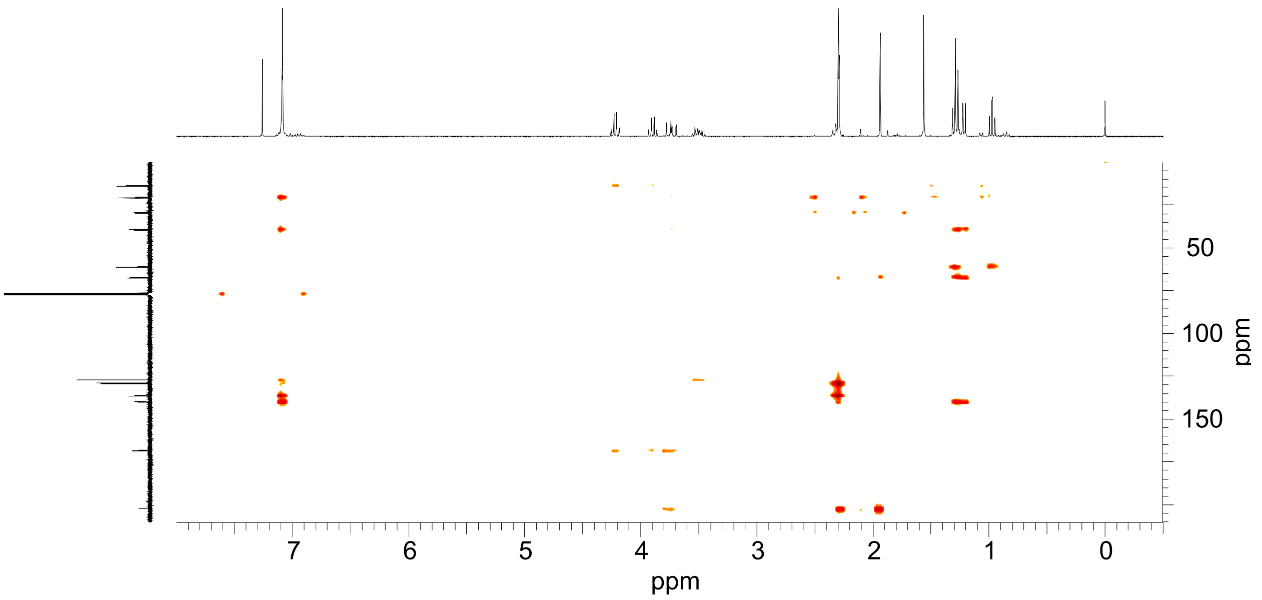

Figure S4. HMBC NMR of compound **3a** in $CDCl_3$.

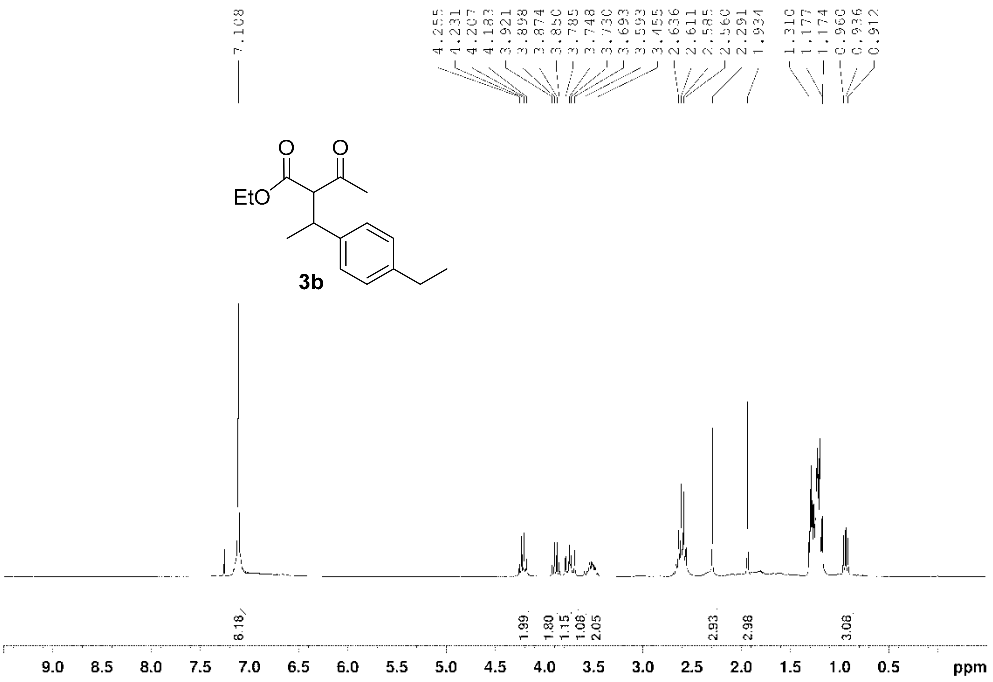

Figure S5. ^1H NMR of compound **3b** in $CDCl_3$.

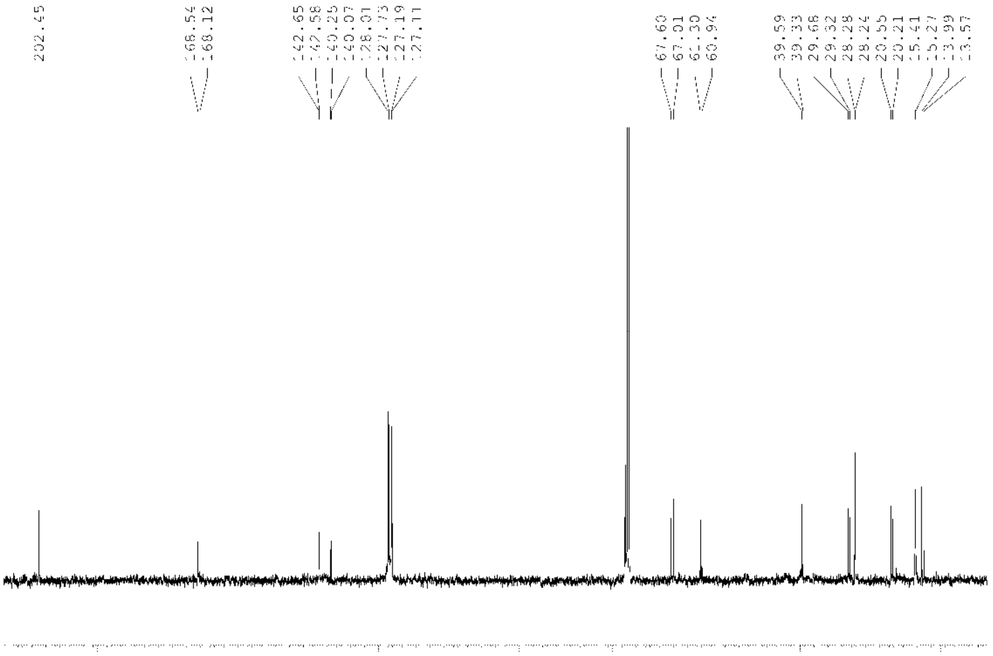

Figure S6. ^{13}C NMR of compound **3b** in $CDCl_3$.

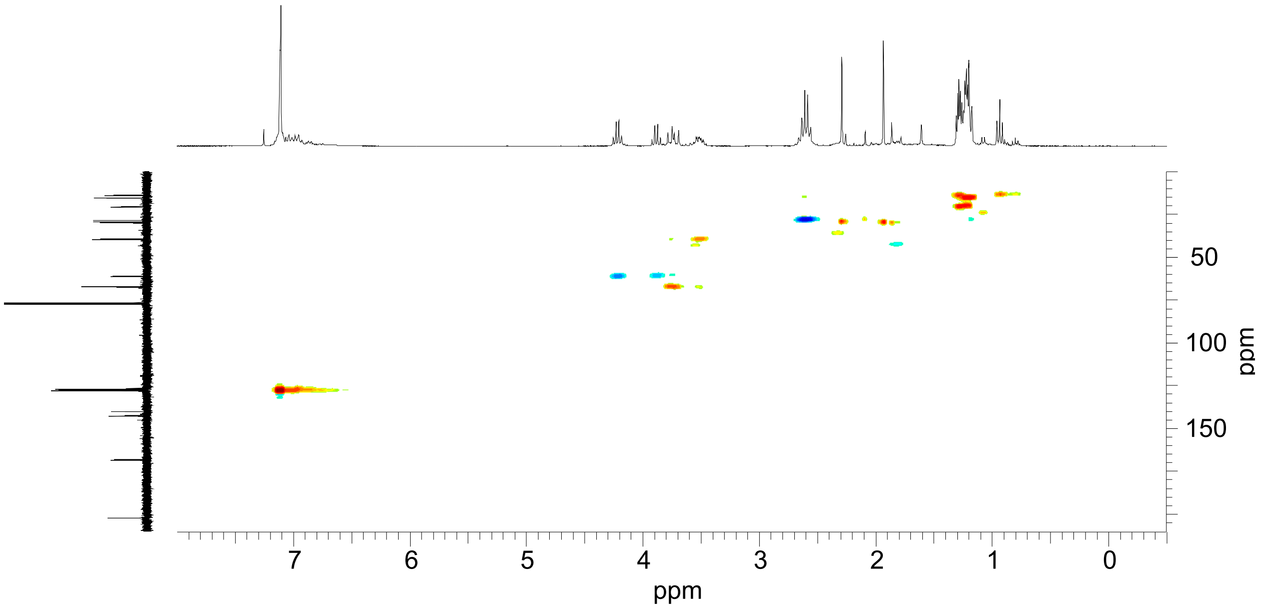

Figure S7. HSQC NMR of compound **3b** in $CDCl_3$.

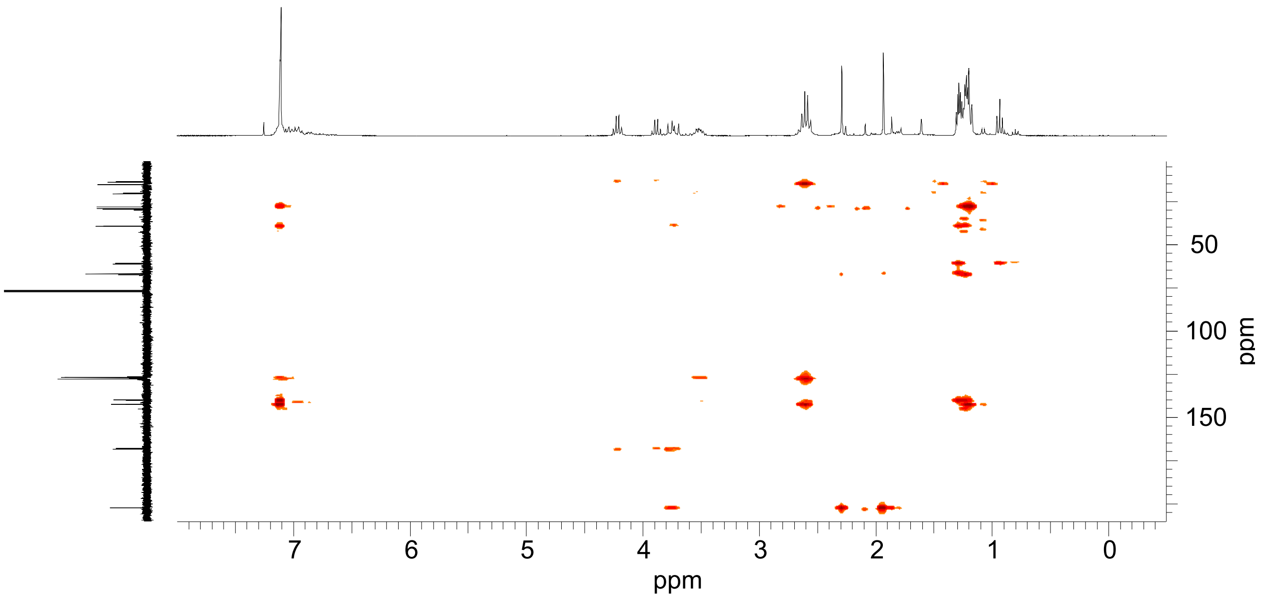

Figure S8. HMBC NMR of compound **3b** in $CDCl_3$.

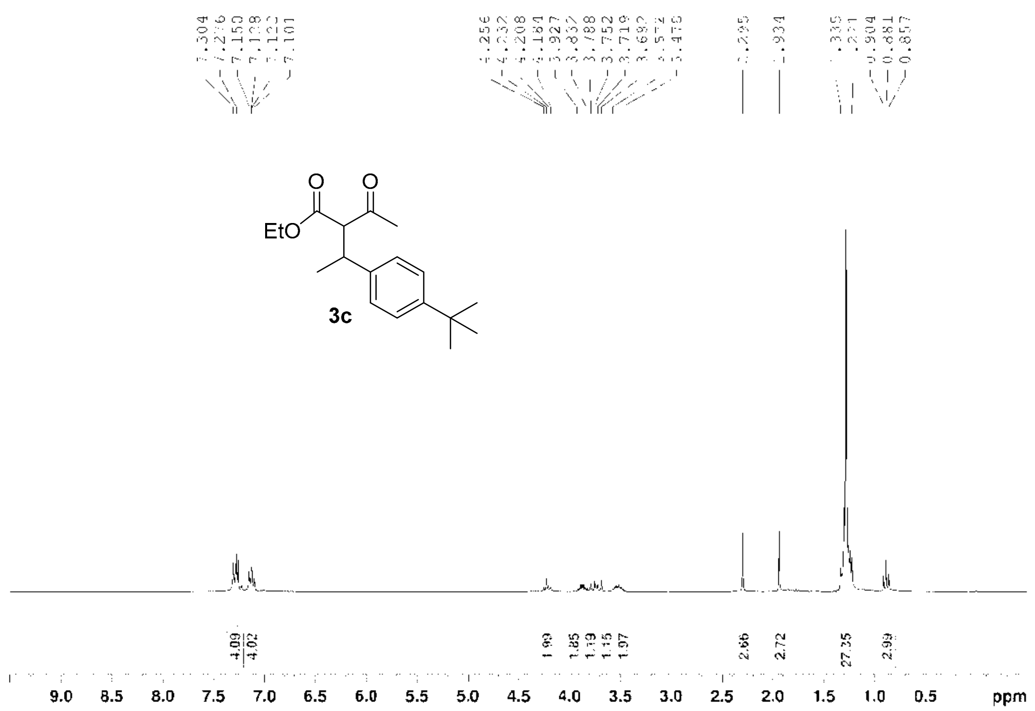

Figure S9. ^{1}H NMR of compound **3c** in $CDCl_3$.

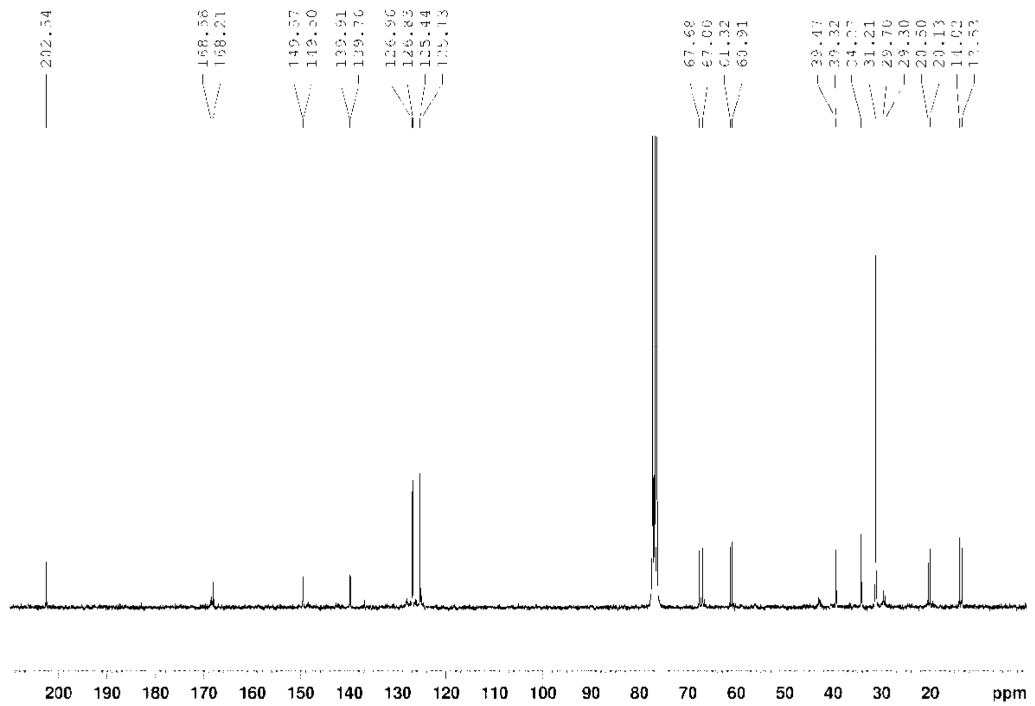

Figure S10. ^{13}C NMR of compound **3c** in $CDCl_3$.

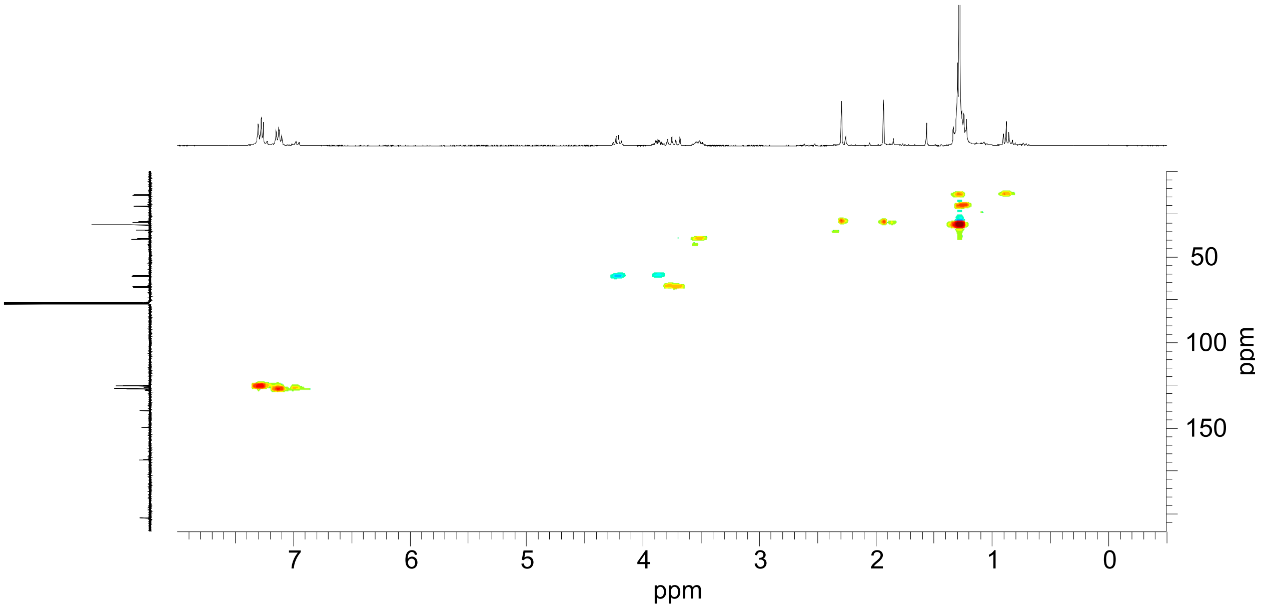

Figure S11. HSQC NMR of compound **3c** in $CDCl_3$.

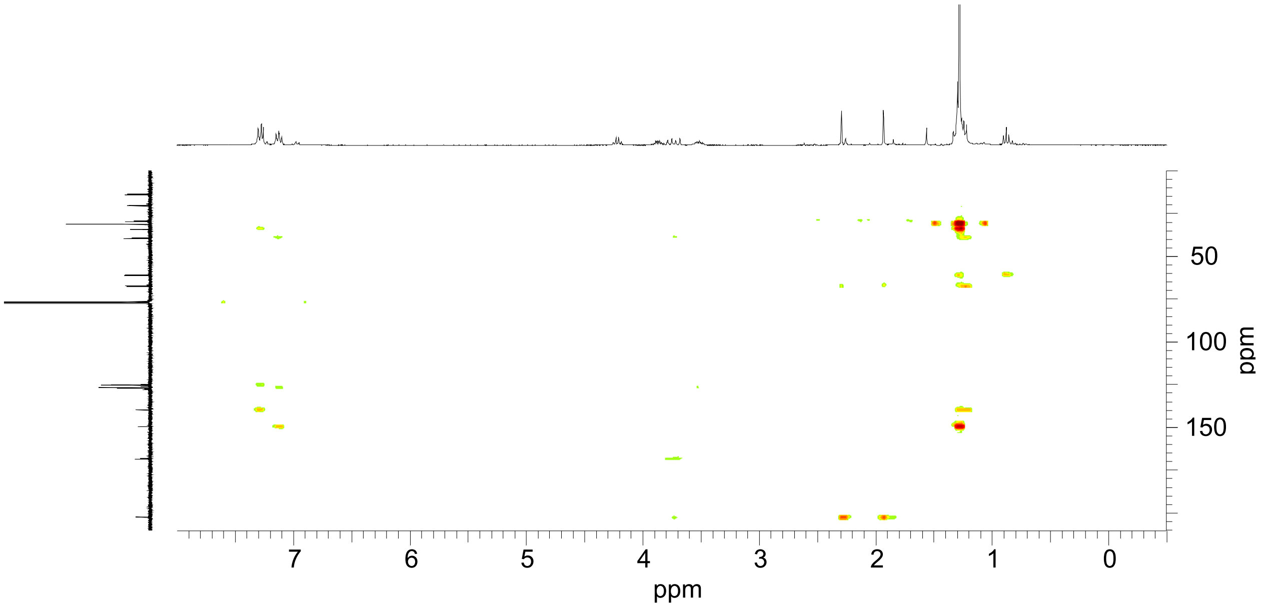

Figure S12. HMBC NMR of compound **3c** in $CDCl_3$.

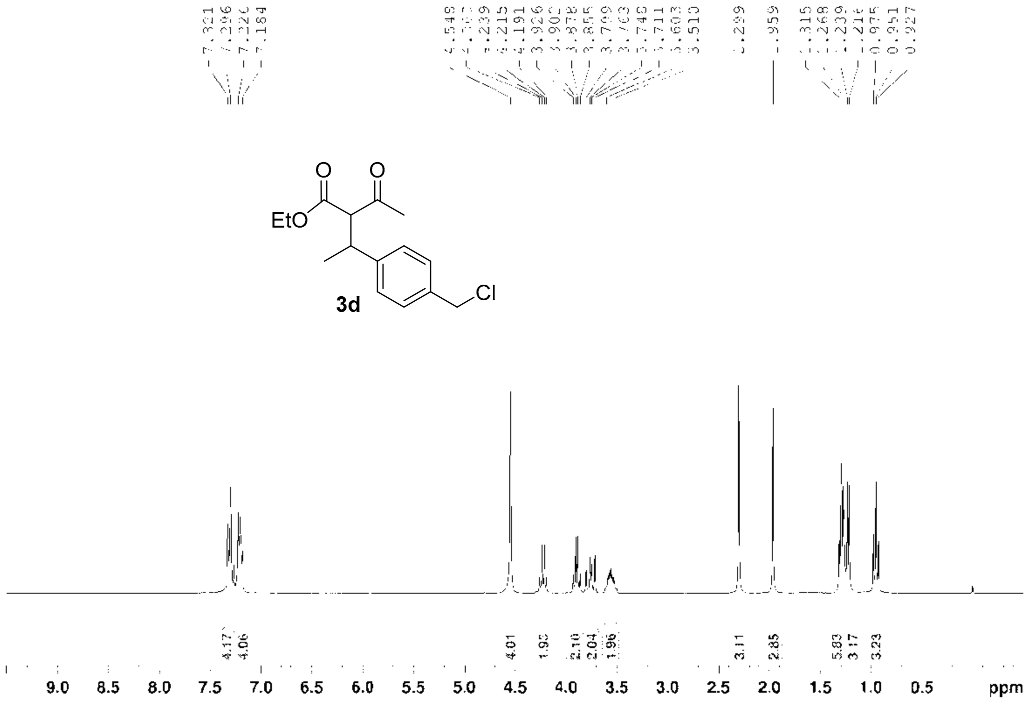

Figure S13. ^{1}H NMR of compound **3d** in $CDCl_3$.

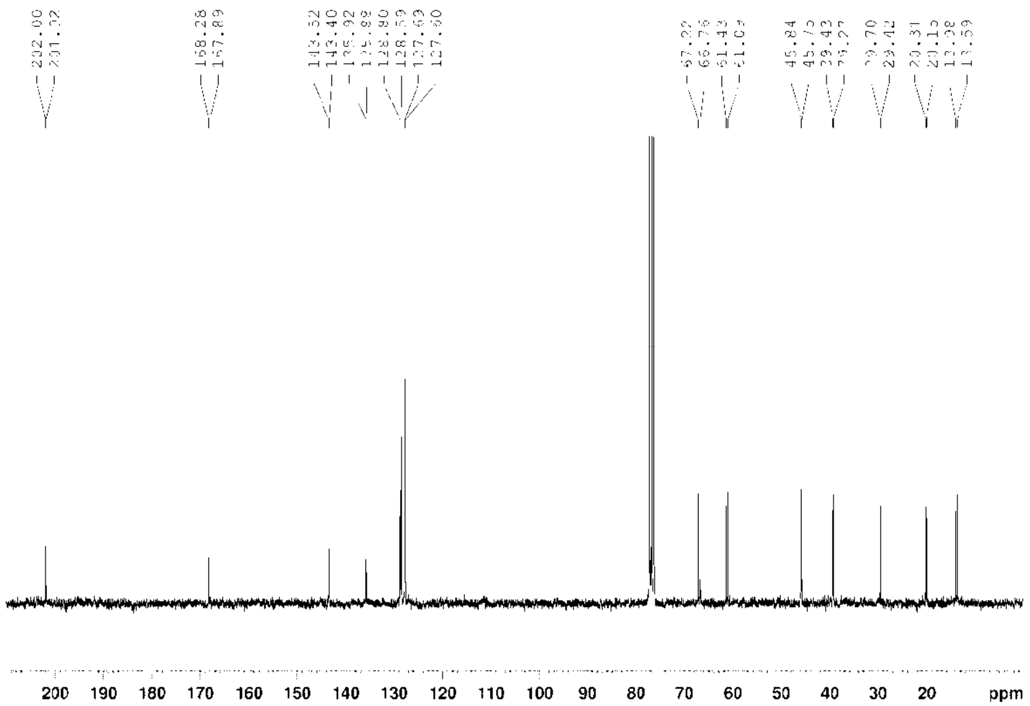

Figure S14. ^{13}C NMR of compound **3d** in $CDCl_3$.

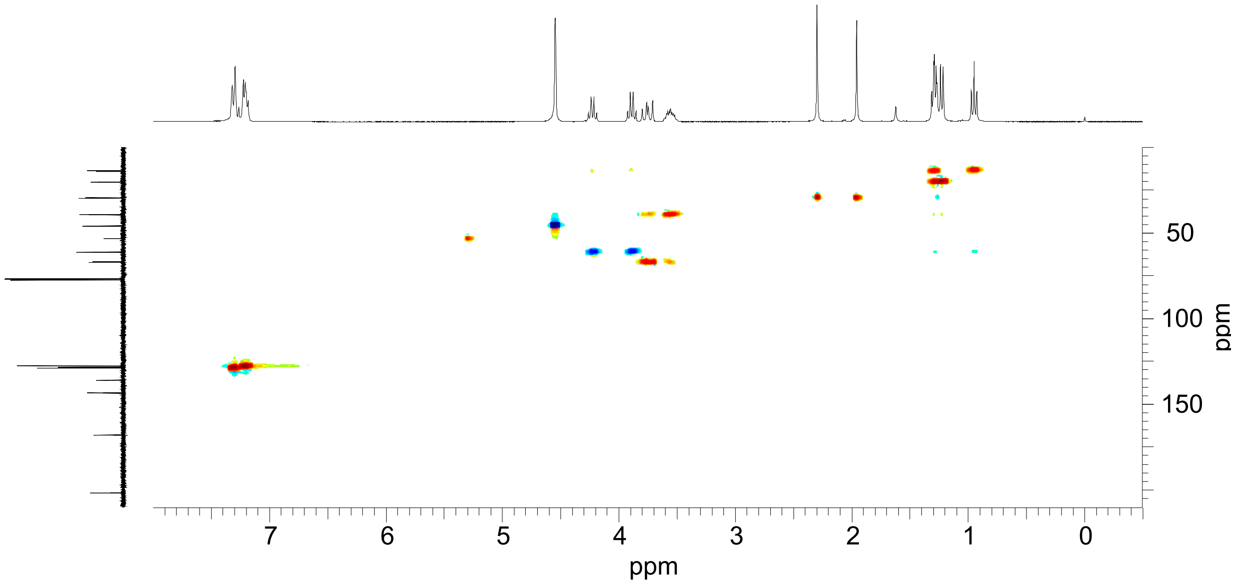

Figure S15. HSQC NMR of compound **3d** in $CDCl_3$.

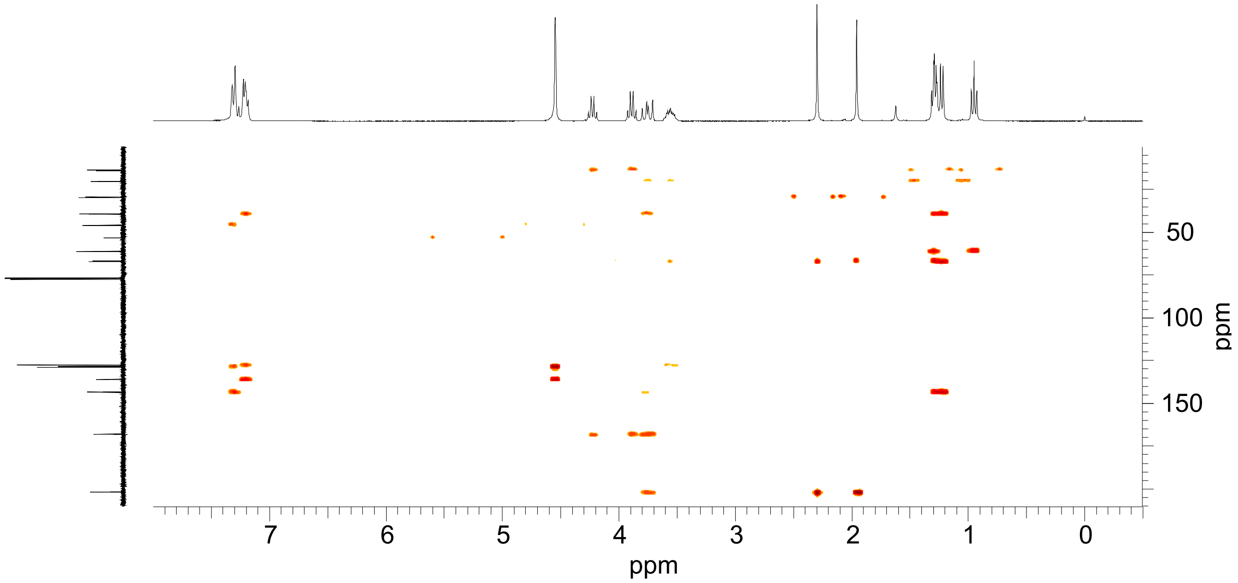

Figure S16. HMBC NMR of compound **3d** in $CDCl_3$.

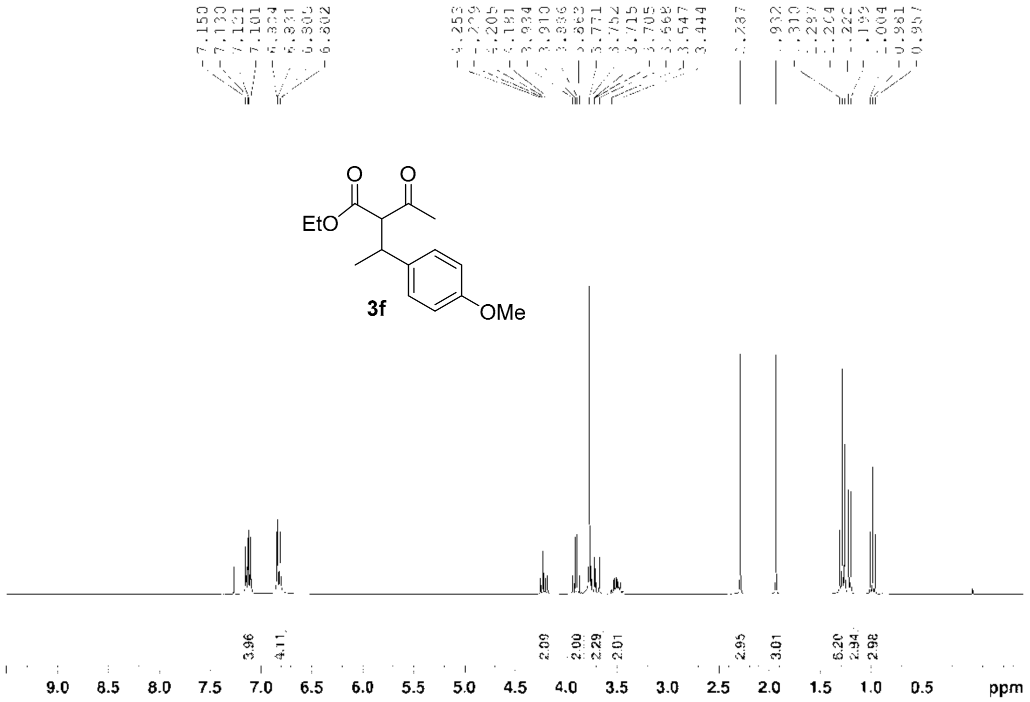

Figure S17. ^{1}H NMR of compound **3f** in $CDCl_3$.

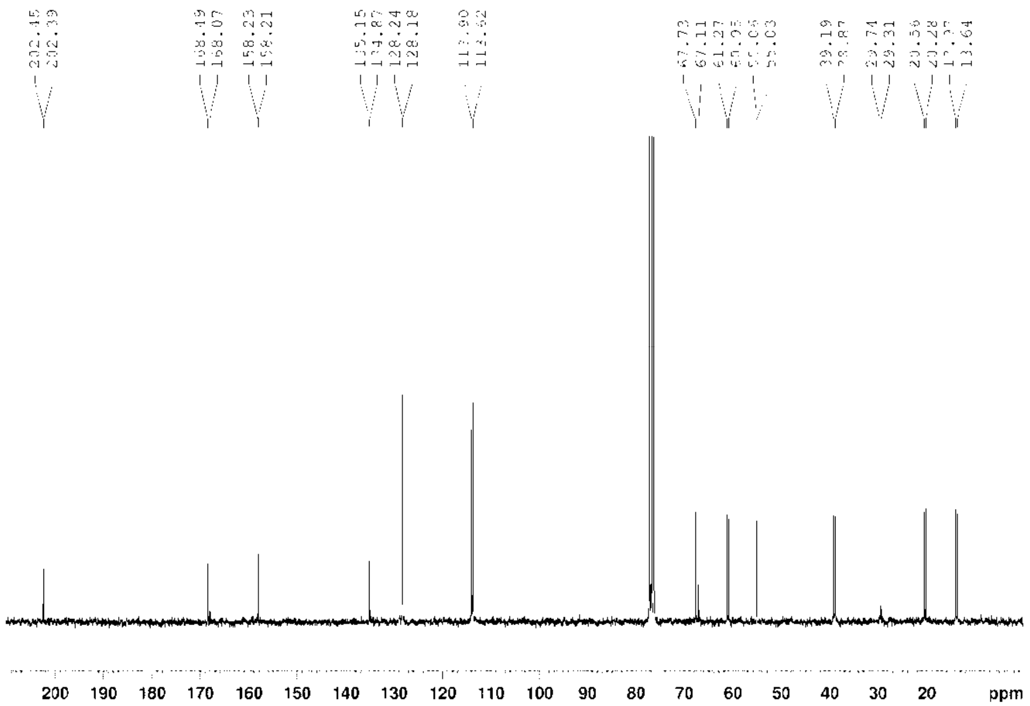

Figure S18. ^{13}C NMR of compound **3f** in $CDCl_3$.

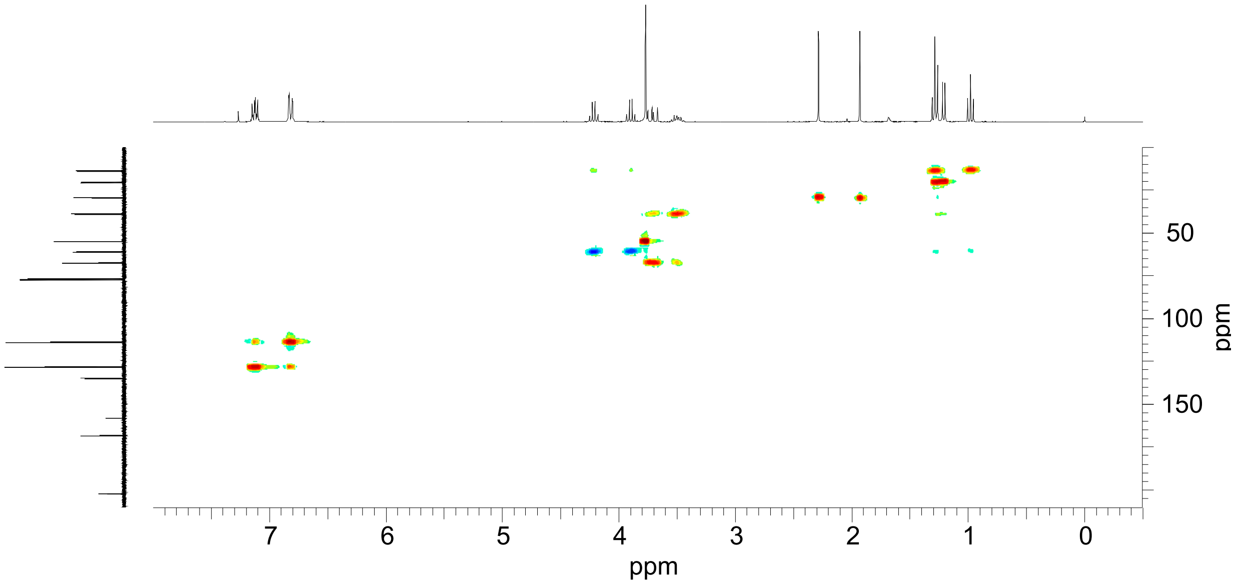

Figure S19. HSQC NMR of compound **3f** in $CDCl_3$.

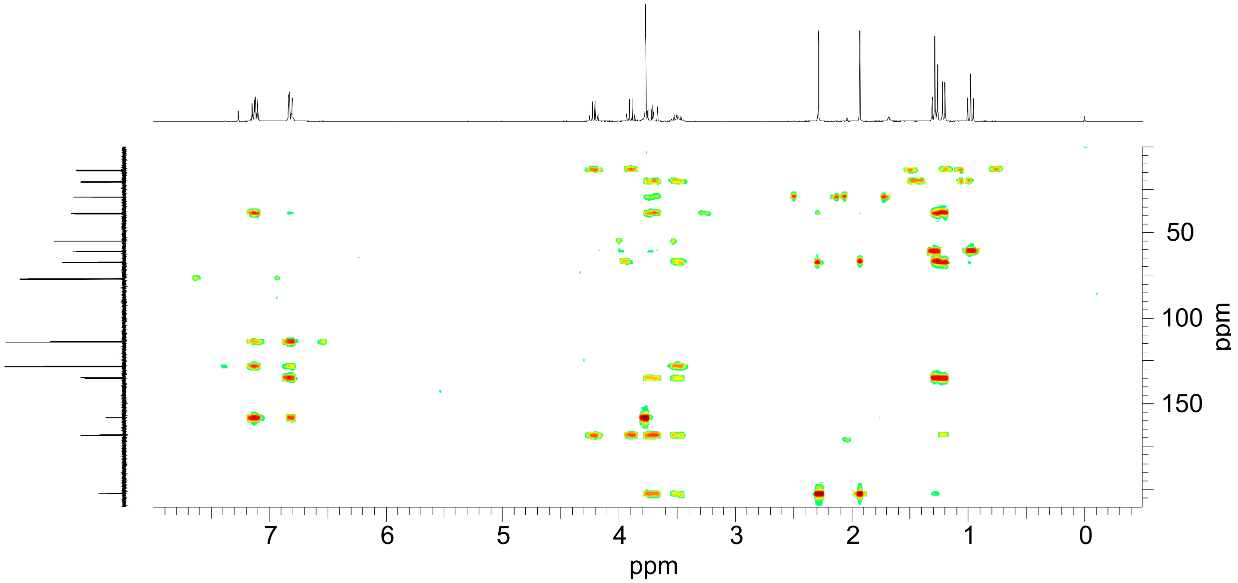

Figure S20. HMBC NMR of compound **3f** in $CDCl_3$.
